# Exploring health practitioners’ acceptability of a prospective semi-quantitative pfHRP2 device to define severe malaria in the Democratic Republic of Congo

**DOI:** 10.1186/s12936-015-0963-1

**Published:** 2015-12-15

**Authors:** Freek de Haan, Marie A. Onyamboko, Caterina I. Fanello, Charles J. Woodrow, Yoel Lubell, Wouter P. C. Boon, Arjen M. Dondorp

**Affiliations:** Innovation Studies Group, Copernicus Institute, Utrecht University, Utrecht, The Netherlands; Kinshasa School of Public Health, Kinshasa, Democratic Republic of Congo; Centre for Tropical Medicine, Nuffield Department of Medicine, University of Oxford, Oxford, UK; Mahidol-Oxford Tropical Medicine Research Unit, Faculty of Tropical Medicine, Mahidol University, Bangkok, Thailand

**Keywords:** Plasma pfHRP2, Diagnostic innovation, Acceptability, Semi-quantitative pfHRP2 device

## Abstract

**Background:**

A rapid diagnostic tool is being developed to discern severely ill children with severe malaria from children who are ill with alternative febrile diseases but have coincidental peripheral blood parasitaemia. The device semi-quantitatively measures plasma pfHRP2 and has the potential to reduce mortality in children with severe febrile illnesses by improving diagnosis. The aim of this study is to identify contributing and inhibiting factors that affect healthcare practitioners’ acceptability of this prospective diagnostic device in a high malaria transmission setting in the Democratic Republic of Congo.

**Methods:**

Data were collected qualitatively by conducting semi-structured interviews with a purposeful sample of health professionals in Kinshasa, capital of Democratic Republic of Congo. In total, 11 interviews were held with professionals at four different institutes.

**Results:**

Four key findings emerged: (1) Congolese practitioners perceive the semi-quantitative pfHRP2 device as a welcome intervention as they recognize the limited reliability of their current diagnostic and therapeutic approaches to severe febrile illnesses; (2) compatibility of the semi-quantitative pfHRP2 device with clinical equipment and competences of Congolese health practitioners is considered to be limited, especially in rural settings; (3) a formal training programme is crucial for correct understanding and application of the semi-quantitative pfHRP2 device; and, (4) provision of evidence to practitioners, and support from health authorities would be important to establish confidence in the semi-quantitative pfHRP2 device.

**Conclusions:**

Congolese practitioners perceive the prospective semi-quantitative pfHRP2 device as a welcome addition to their clinical equipment. The device could improve current diagnostic work-up of severe febrile illness, which might consequently improve treatment choices. However, despite this recognized potential, several hurdles and drivers need to be taken into account when implementing this device in DR Congo.

## Background

It is estimated that one in every ten newborns in the sub-Sahara African region dies before reaching the age of five [1]. A high burden of infectious and other childhood illnesses, in combination with underperforming healthcare systems, lies at the root of this alarming number. One major problem relates to the diagnosis of severe febrile illnesses. Clinical diagnosis is difficult because symptoms of different diseases overlap, and adequate diagnostic tools are frequently unavailable [2]. This problem is particularly prevalent in the context of falciparum malaria. Malaria is one of the major causes of child mortality in sub-Saharan Africa, with an estimated 468,000 malaria attributed deaths under the age of 5 years in 2012 alone [3]. Clinical symptoms of a malaria infection are not specific and there is significant overtreatment in children with a clinical diagnosis of malaria [4]. Recognition of this problem is reflected in WHO guidelines stressing the importance of confirming suspected malaria either via microscopy or malaria rapid diagnostic test (RDT) before administering treatment [5]. This paradigm of evidence-based treatment improved management of both malaria and non-malarial febrile illnesses in the region by enabling practitioners to administer more appropriate medication with increased confidence [3].

However, correct diagnostic work-up of febrile ill children in medium to high falciparum malaria zones remains challenging. In such areas, febrile symptoms are often still equated with malaria, and treated accordingly, even when RDT results are negative [4, 6]. Another, more nuanced problem associated with correct malaria diagnosis is represented by febrile ill children who have laboratory or RDT confirmed malaria, but where the malaria infection is not causing the febrile episode. Instead, the illness is concomitant to an alternative but undetected infection whilst presence of the malaria parasites is coincidental. This is frequent in high transmission areas where children develop immunity to *Plasmodiumfalciparum* parasites during the early years of life or where they experience an extended period of parasite positivity after effective treatment of a recent malaria episode [7, 8]. These semi-immune children can tolerate parasitaemia, but remain asymptomatic despite carrying parasite burdens detectable with microscopy or RDT. Currently, no available reliable diagnostic method distinguishes between children who suffer from severe malaria and children with severe non-malarial febrile illness and a co-incidental episode of uncomplicated malaria. When malaria is detected in a febrile child, the health practitioner will obviously begin anti-malarial treatment, neglecting the high a priori chance that such a child is parasitaemic due to background prevalence.

Research shows that up to 20 % of children presenting with severe febrile illness and a positive malaria slide have an alternative diagnosis, often in the form of invasive bacterial sepsis [4, 9–13]. This causes overdiagnosis of malaria, whereas the non-malarial but potentially fatal febrile illness often remains undiscovered and untreated. An innovative approach to this problem relates to the discovery of plasma *P. falciparum* histidine-rich protein 2 (pfHRP2) concentrations as a measure for total parasite burden, including the sequestered parasite biomass not detectable in the peripheral blood [14]. Plasma pfHRP2 strongly correlates with malaria disease severity and low values make *P. falciparum* as a cause of severe disease unlikely as was demonstrated by research [15, 16], with similar findings being obtained across varying settings [17–20]. No other biomarker matches plasma pfHRP2 in this regard. Additional support for low plasma pfHRP2 as indicator for the high likelihood of an alternative diagnosis than just falciparum malaria causing the severe febrile illness comes from an observation made in the large trials comparing parenteral artesunate with quinine for the treatment of severe malaria (AQUAMAT and SEAQUAMAT) [21, 22]. The lower benefit of artesunate over quinine in African children compared to Asian adult patients is entirely explained by lack of additional advantage in children in the lowest tertile of plasma pfHRP2 levels, where misdiagnosis is likely [23].

Understanding of the diagnostic potential of plasma pfHRP2 prompted efforts to translate this approach into a point-of-care diagnostic device. In the current paper, this emerging tool will be referred to as semi-quantitative pfHRP2 device. The semi-quantitative pfHRP2 device measures plasma pfHRP2 levels and will be applicable to rapidly indicate the likelihood of malaria being the underlying cause of severe febrile illness in parasitaemic African children. Since direct and swift intervention is essential to prevent mortality in these cases, the semi-quantitative pfHRP2 device would be particularly suitable for emergency management, as an additional step in the diagnostic work-up. The device is being developed in a point-of-care, lateral-flow design similar to RDT, including a readout where coloured lines will appear according to thresholds in parasite concentrations. Those concentrations will be measured semi-quantitatively, with a high cut-off measure above which *P. falciparum* is highly likely the cause of the presenting severe febrile illness, requiring prompt parenteral anti-malarial treatment. In addition, there will be a low cut-off measure, below which the episode of severe febrile illness is highly likely caused by an alternative diagnosis while presence of *P. falciparum* parasites is not causing complications. This situation would warrant further investigation with additional diagnostic assessment and alternative treatment, including targeted antibiotics [15, 16].

Even though the diagnostic value of plasma pfHRP2 is promising, it is not clear whether health practitioners in high malaria transmission settings would embrace this prospective device. Important factors for successful adoption of—and adherence to—this diagnostic innovation are the extent to which the test fits within the healthcare system and socio-behavioural needs are addressed [24]. This study is a first exploration aiming to identify relevant contributing and inhibiting factors affecting acceptability of the semi-quantitative pfHRP2 device by its intended end-users in the Democratic Republic of Congo (DR Congo).

## Methods

### Study area

DR Congo was selected as the site of study because its child mortality rates and malaria prevalence are amongst the highest in the world [25]. This makes DR Congo an important target area for the semi-quantitative pfHRP2 device. The country is located in central Africa and has an estimated population of 75.5 million [26]. The official language is French, a heritage from the Belgium colonization period, although various other languages are being spoken in different districts. The capital of DR Congo, Kinshasa, is situated in the west and houses circa 8.6 million people [26]. The Human Development Index ranks DR Congo as one of the least developed countries in the world with a GNI per capita of 300 USD and 80 % of the population living on less than 1 USD a day [27]. Congolese life expectancy is 50 years, and one in seven children die before their fifth birthday. The estimated fraction of malaria in under-five mortality is 19 % [28]. Availability of anti-malarial medication and of diagnostic tests is considered to be high in urban areas compared to rural ones in DR Congo. The 2013 surveys by ACTWatch in Kinshasa indicate that anti-malarial medication is available in nearly all private and public health facilities and drug outlets. Chances of receiving quality-assured ACT therapy is highest in Kinshasa’s public health facilities, with an estimated 50 %. Diagnostic tests are widely available in both private and public health facilities; over 90 % of Kinshasa’s health facilities supplying anti-malarial medication also have malaria diagnostics available [29].

The Congolese public healthcare system is formed around 516 health zones, with a total of 393 reference hospitals, and 8266 lower-level health facilities [26]. There is no public insurance or remuneration system, and healthcare costs are usually ‘out-of-pocket’ expenses paid by patients. Malaria transmission is high and stable in most of the country [3]. Malaria policy in DR Congo is coordinated by the National Malaria Control Programme (NMCP).

### Data collection and analysis

Data were collected qualitatively by conducting semi-structured in-depth interviews with a purposeful sample of health professionals in Kinshasa. A total of 11 interviews were conducted with: (1) three nurses from a public hospital in Kinshasa; (2) three local malaria researchers; (3) four medical specialists connected to the local academic hospital (an entomologist, a paediatrician, a tropical medicine specialist, an internist); and, (4) one NMCP representative.

Interviews were conducted face-to-face, and were recorded with consent of each interviewee. These recordings were supplemented with auxiliary notes made during the interview. Concepts of adoption theory [30] and behaviour change theory [31] were used to inspire focal topics for investigation in data collection. Adoption theory allowed for assessing technology specific attributes of the semi-quantitative pfHRP2 device. Behaviour change theory enabled approaching the adoption decision from an intrinsic, personal point of view. With some respondents, not all concepts were discussed since they were not relevant to their area of expertise. Prior to each interview, respondents were given an information sheet, which provided an explanation of the semi-quantitative pfHRP2 device including a schematic visualization of its proposed position in the diagnostic work-up. Information sheets were available in both French and English. Two of the interviews were held in English and the other nine in French. The French interviews were conducted with assistance from a professional translator experienced with healthcare projects. The translator was thoroughly lectured about the project, interview questions were reversely translated and a practice interview was held to minimize the interpretative bias. After the interviews, translator and researcher jointly made transcripts for each interview. These transcripts were then line-to-line coded by the researcher using the theory-driven concepts. These focal concepts were complemented by taking into account insights that emerged from the interviews, creating new codes. Subsequently, pieces of text with similar content were grouped together and concepts with higher levels of abstraction were formed by a process of induction. In this way, both contrasting explanations and recurring themes were captured from data.

## Results

Four key contributing factors were identified in the interviews with Congolese health professionals: (1) the semi-quantitative pfHRP2 device is perceived as an intervention with potential to improve management of children with febrile illness; (2) the device is considered to have limited compatibility with equipment and competences of Congolese practitioners, especially of those active in rural settings; (3) a formal training programme is crucial for correct application and interpretation of the semi-quantitative pfHRP2 device; and, (4) credible evidence provided to practitioners, but supported by health authorities, would establish confidence in the device. These key factors are now presented in more detail, with supporting quotes to illustrate or exemplify findings.

### Perceived advantage of the semi-quantitative pfHRP2 device over current febrile illness diagnostic practices

The problem of distinguishing children who are ill because of malaria from children with a non-malaria febrile illness and coincidental parasites in their blood was recognized by the respondents. Due to the current lack of a tool to make this distinction, the prospective semi-quantitative pfHRP2 device was considered a welcome addition to their clinical equipment. This position was exemplified by respondent 6:

“When immune children are sick and tested for malaria, they will test positive. But they may have another type of disease. It would be good to have a rapid test for this. Very many people will get the clinical diagnosis of malaria but are in fact sick with another disease.”

Respondents mentioned three practices which they know are currently employed to deal with the possibility of a co-existing non-malarial febrile illness after malaria is diagnostically detected. The first is to scrutinize each febrile patient for symptoms specific to a febrile disease prevalent in the area, and base treatment on this hypothetical judgment. However, respondents consider this symptomatic search for non-malarial febrile illnesses as impractical since most febrile symptoms are not specific to one disease. As an alternative to this method, the prescription of anti-malarial medication to all severe febrile patients whilst initially neglecting the possibility of a co-existing alternative febrile illness was proposed. This approach was also considered inappropriate for emergency management since the possibly life-threatening non-malarial febrile illness would not be restrained in these cases. The third suggested option is to always supplement anti-malarial medication with antibiotics. This approach was, however, judged as disadvantageous since scarce medication may be wasted.

Throughout the interviews it was repeatedly emphasized that availability of a semi-quantitative pfHRP2 device would open up a fourth option. The addition of an extra diagnostic step to indicate the likeliness of a co-existing febrile illness after malaria is detected would allow health practitioners to improve prescription of prompt and suitable medication with more confidence. Respondent 4, for example, voiced this appraisal:

“To me a test to show that the illness is caused by malaria would be a huge improvement. Because with this rapid test you would have evidence-based back-up […]. It should then be a very simple test that can be used in the field”.

Respondent 1 complemented this by stating that:

“With such a diagnostic, we can treat patients for other diseases than malaria, [diseases] in which we now lose time because we can only treat symptoms while we do not know what it is.”

Since the semi-quantitative pfHRP2 device is being developed as a follow-up step in the diagnostic work-up, its application would inevitably imply increased clinical costs per febrile illness episode. Respondents indicated that this could be a barrier to adoption. However, when effectiveness is proven by either reduced mortality or more appropriately targeted treatment, willingness to pay is expected to increase. Additionally, inclusion in a subsidy programme, as is currently the case with RDT in DR Congo, was put forward as an effective way of overcoming the financial barrier to adoption by reducing costs:

“RDTs in the country are now subsidized. They are free in the public sector. So this test should also be free or really, really cheap. A commercial price would probably be too expensive. But if you have shown that your test really helps, then people are willing to pay” (Respondent 4).

### Perceived compatibility semi-quantitative pfHRP2 device with current febrile illness management

To effectively incorporate the semi-quantitative pfHRP2 device into febrile illness management, practitioners must have the ability to interpret the test results and act accordingly. Evidence that this cannot be assumed emerged in the form of three compatibility-related adoption barriers.

First, it came to light that practitioners regularly lack awareness of the disease range in their organizations’ locality, and that fever is still equated with malaria. They lack proficiency to deal with the possibility of an alternative febrile illness. Respondent 4 explained that

“….when the illness is something else [not malaria], they are not prepared for that. They [physicians] are not trained to manage these cases. You would expect that they are trained to look for signs of other diseases in the case of a negative test, but they are not”.

This problem was not associated with well-educated healthcare professionals who are usually clustered in and around urban areas. Rather, respondents primarily pointed to practitioners in rural areas of DR Congo, since:

“Given the dimensions and size of Congo, and with all problems here including the war, competent people do not like travelling to the inner country. They prefer living in the city. Many of the practitioners living in small towns have no experience with diagnostics. We should not bring sophisticated techniques to health workers in the inland; because they are not competent” (Respondent 5).

The second barrier concerns the possibility to follow-up on a negative test result of the semi-quantitative pfHRP2 device. Respondents mentioned that Congolese hospitals regularly lack tools to identify both malaria and non-malarial febrile illnesses. Respondent 3, for example, attested that

“In case of illness, the nurses will use some clinical signs and they can give children drugs to repress fever and some antibiotics. Because there are often no tests available to discover which disease may join malaria”.

When there is no practice available to conduct a suitable follow-up diagnostic work-up, a negative semi-quantitative pfHRP2 test result would be an empty step within the process. Such a result would merely present malaria as an unlikely cause of the severe febrile illness, but the actual cause would remain unidentified and suitable treatment would remain abstruse.

Third, a regular stock-out of appropriate medication was considered an important obstruction to adopt the semi-quantitative pfHRP2 device. Respondent 3 evinced this with observations made in Bolenge town:

[Respondent describes a fever epidemic where, many children tested positive for malaria]. “And the result was that 80 children were suffering from malaria or another fever. But there were no more drugs to provide; all were finished.”

Whereas the unavailability of a diagnostic can be replaced by switching to presumptive diagnosis, alternatives for medication stock-out hardly exist. The only suggested options were referring the patient to another facility or acquiring medication in a private drug shop. The latter case was perceived unattractive since in these situations prices can increase ten-fold according to one respondent. Again, the problem of diagnostic and medication stock-out was mainly associated with isolated rural areas.

### Application and interpretation of the semi-quantitative pfHRP2 device

The semi-quantitative pfHRP2 device will be developed as a rapid, point-of-care test design using a drop of blood on an indicator strip (lateral flow design) whereupon the plasma pfHRP2 level will be displayed on a read-out. In the hospitals under study, practitioners conveyed that they are familiar with similar tests, such as lateral flow format RDT. They declared that this familiarity brought them to perceive themselves capable of rapidly learning its application. It was stressed that a practical training session is nonetheless required every time a new product is introduced and the semi-quantitative pfHRP2 device would not represent an exception.

“An introduction programme should be similar as what was done with the RDT. So when there is a new test, the first thing is to have a practical training. Someone has to show the specific techniques and how to use the product” (Respondent 11).

In contrast to the practical application, respondents did anticipate complications regarding correct interpretation of test outcomes. Whereas conventional diagnostic devices usually indicate presence of an illness, the semi-quantitative pfHRP2 device will provide more nuanced information on the probability of the underlying cause of illness. Here, a more elaborate and in-depth training programme was thought to be required, as was exemplified by respondent 2:

“An interpretative training would be more difficult with this new test than with the RDT because they would have to understand what could be the different stages of the test and what exactly happens in each of these stages”.

It was suggested that interpretative complexity of the test results could be further reduced by practicing how to respond to each possible scenario of test-outcomes: “[…] in the training programme they should also include projects on how to interpret the results; what to do in case of a positive and in case of a negative diagnosis” (Respondent 4).

Finally, the importance of a clear definition of the target patient population was deemed crucial to establish understanding in the semi-quantitative pfHRP2 device.

### Evidence provision and experimentation with semi-quantitative pfHRP2 device

Respondents indicated that their practices are largely dependent on healthcare policy recommendations. Inclusion of the semi-quantitative pfHRP2 device in treatment guidelines from a governmental health authority would be a strong re-inforcement towards adoption. Governmental endorsement, such as the NMCP, was deemed crucial to acceptability. Congolese practitioners would not perceive evidence provided by other parties, such as manufacturers, as reliable.

“People from the Ministry would have to confirm your product, because if you approach them by yourself, people will not believe you. But when they see a member of the Health Ministry joining you, then you have the credibility” (Respondent 2).

One health practitioner expressed that adherence to principles of good governance within governmental authorities is not obvious in DR Congo, and that conflicting interests could affect objective endorsement by members of the NMCP.

As well as the importance of national guidance, respondents emphasized the relevance of taking local interpretations into account. For example, respondents knew of various instances in which practitioners may decide to deviate from national guidelines due to a differing personal judgment. Evidence that the semi-quantitative pfHRP2 device is effective and reliable should therefore not solely be presented to the NMCP, but also to practitioners personally:

“You would need to have the result of clinical trials, which are relevant for both the NMCP and the doctors. Because also doctors need to learn about the accuracy” (Respondent 6).

It was further emphasized that, in order to be perceived as credible, presented evidence should have been obtained from the same respective area as where it is presented, since locally obtained evidence is perceived to be more trustworthy than evidence from elsewhere. Conditions such as disease prevalence and intensity differ per area and evidence from another country or even another locality within DR Congo would not be regarded as credible.

“I insist that all new products must be tested under local conditions. Also within the country we have different circumstances and different cultures” (Respondent 3).

This significance for locally collected evidence, combined with a de facto accreditation by the national government, leads to intense vertical learning but not so much to knowledge transfer between hospitals.

It was furthermore put forward that enabling health practitioners to experiment with the semi-quantitative pfHRP2 device would increase their willingness to adopt.

“We should have time to experiment with the techniques and the material of the new test […] After that we have to practice in the hospital, and by doing so, experience is raised and the practice becomes more and more fixed” (Respondent 11).

Whilst practitioners are usually sceptical of new practices, a try-out period would increase their confidence in the device’s effectiveness. Positive outcomes are valued during such trial period since a respondent stated that attitudes towards the device would be influenced negatively if the test did not work well.

## Discussion

The paradigm shift towards parasite-based diagnosis of malaria has enhanced recognition of the importance of integrating malaria management with management of other febrile illnesses, as also propagated in previous studies from Ghana and Zanzibar [32, 33]. Usage of the semi-quantitative pfHRP2 device could be an additional step in that direction with particular relevance to the refinement of management in severe disease. A more nuanced insight into the cause of a severe febrile illness episode, as obtainable by this prospective device would enable practitioners to further improve management of both malaria and non-malarial febrile illnesses. Severe febrile illness caused by either malaria or invasive bacterial infections or by a combination of these poses an important problem for the treating health practitioner. As reviewed by Church and Maitland, one-third of all deaths from severe malaria occur in children with bacterial co-infection and the main pathogens involved are enteric gram-negative organisms and non-typhoid salmonellae [34]. In addition to low plasma pfHRP2, both recent malaria (defined as whole blood pfHRP2-based RDT positive, but malaria slide negative) and hyperparasitaemia have been shown to be a risk factors for concomitant bacteraemia complicating malaria [9, 13]. The benefit of an additional simple test to distinguish better between the different syndromes would be a core advantage over other currently available febrile illness management algorithms. This was recognized by Congolese practitioners given that respondents explicitly mentioned that the semi-quantitative pfHRP2 device would be a welcome addition to their clinical equipment. Even so, they are neither likely to initiate adoption themselves nor be open to adoption when approached by a commercial manufacturer. Rather, support of the Health Ministry implicit in treatment guidelines was emphasized as a requisite for embracing the semi-quantitative pfHRP2 device. Recommendation of the device by governmental authorities such as the NMCP is perceived as proving reliability whilst lack of support reduces both accessibility and visibility. This perceived significance for central coordination is noteworthy, since a lack of trust in the national public healthcare system was also voiced.

A second finding is that evidence of the effectiveness of the semi-quantitative pfHRP2 device should be presented to practitioners personally rather than solely to the NMCP. Even though inclusion in NMCP guidelines implies that a test is useful, practitioners also exercise a degree of autonomy and personal judgment. The more convinced they are that the semi-quantitative pfHRP2 device bears the purported clinical benefits, the more willing they will be to adopt it. A further finding of interest is the preference for local demonstration of the advantages of a novel tool. Practitioners are sceptical towards evidence gathered in other healthcare sites than those in their own region. It was, for example, explicitly mentioned that evidence of effectiveness from Zambia would be perceived close to worthless in DR Congo, but also within the country evidence should, according to respondents, be collected and distributed locally. In terms of evidence gathering and knowledge production, this study contrasts with findings from Chandler in Ghana, where it was proposed that social learning processes between practitioners are essential for reaching the full potential of RDT [35]. In the case of the semi-quantitative pfHRP2 device in DR Congo, respondents expected learning processes to be more top–down and centrally coordinated instead of horizontal. The importance of evidence being locally produced and distributed is not yet represented in published literature. It is important to realize that recognition of the usefulness or importance of an additional diagnosis tool does not imply necessarily that the test will change practitioners’ decision-making. Different studies have shown that adherence to point-of-care malaria tests often are considerably low, even when training sessions are organized to emphasize the importance of appropriate follow-up behaviour [36, 37]. This problem is especially related to malaria overdiagnosis where practitioners regularly prescribe malaria treatment despite negative diagnosis [36]. The culture of limited consideration of negative test results could also be a threat to reaching full potential of the semi-quantitative pfHRP2 device.

Thirdly, respondents deemed themselves capable of learning quickly how to utilize the device. Their experience with similar test designs, such as RDT, is expected to accelerate their attainment of the required technical skills. Nonetheless, respondents emphasized the importance of a formal training programme to become familiar with the practical appliance of the device and its proposed position in the broader context of febrile illness management. The significance of formal training sessions, including provision of information on appropriate follow-up of negative cases, has been stressed extensively in the context of implementation of RDT [35, 38]. These adoption factors also seem to be relevant in the case of the semi-quantitative pfHRP2 device. Furthermore, one study emphasized the importance of technical supervision, consistent training messages and a continuing quality-control system to build and sustain confidence in RDT in the Solomon Islands [38]. The current study is consistent with these findings, but extends the list by stressing the significance role of governmental authorities in providing training messages and the possibility for practitioners to try out the semi-quantitative pfHRP2 device prior to adoption.

Despite the recognized potential of the semi-quantitative pfHRP2 device in the Congolese healthcare system, two major adoption barriers emerged from this study. The first concerns the extra costs associated with adoption. Since public insurance and reimbursement systems are non-existent in DR Congo, healthcare expenses are usually ‘out-of-pocket’ payments by patients, and practitioners are directly dependent on these payments for their own income. Due to this payment system, offering the semi-quantitative pfHRP2 device at a commercial price was considered likely to hamper adoption. Respondents were cautious in offering concrete price indications as ‘willingness to pay’ is largely determined by specifications such as test specificity and sensitivity and also depends on the price of related diagnostic tools and follow-up medication. A previous study in Tanzania has shown that cost-effectiveness of malaria diagnostics depends on consistent adherence to test results and also correlates with transmission rates [37]. A suitable follow-up for the current study would be cost-effectiveness and ‘willingness to pay’ studies taking into account such elements for the semi-quantitative pfHRP2 device.

The second barrier relates to the compatibility of the semi-quantitative pfHRP2 device with current febrile illness management practices. A prerequisite for usefulness of the device is that its users have relevant clinical resources and personal capabilities to manage the device in this broader febrile disease context. This is expected to be problematic in DR Congo since knowledge of the local ‘illness range’ regularly appears absent, and the additional information obtained using the semi-quantitative pfHRP2 device is expected to be complex. Moreover, availability of diagnostics to detect non-malarial febrile illnesses is mostly absent in the country, as well as access to appropriate medication. This would compromise the benefit of the semi-quantitative pfHRP2 device for the diagnostic work-up. These challenges are primarily associated with isolated rural areas in the Congolese inland. Especially in the rural settings, there is a consistent lack of diagnostic and medical resources whilst those localities also suffer from a shortage of competent and educated practitioners. In urban areas, resources are more clustered and there is usually a possibility to refer a patient to another facility with more expertise and clinical resources [2]. The relevance of compatibility issues to rural hospitals compared to urban equivalents was pointed out by respondents in the current research.

Some limitations of the study must be taken in consideration. Firstly, the study was purely qualitative in nature and thus cannot provide insight into the degree to which adoption factors are relevant. Secondly, even though efforts were made to minimize interpretative bias, it is inevitable that certain nuances are lost by intervention of the translator. An important limitation with regard to external validity relates to the fact that the research was conducted in different health centres in one city, Kinshasa. This may imply limited generalizability of their statements and visions to the whole country, which accommodates many cultures and local factors in a large territory. However, a great number of respondents indicated that they have worked in other (rural) localities and therefore this could make their assessment more balanced. Conducting the study within a broader sample of respondents in different hospitals and inclusion of rural hospitals would be an appropriate follow-up step for this research.

## Conclusion

Respondents perceived the emerging semi-quantitative pfHRP2 device as a welcome addition to practitioners’ clinical equipment. The device could improve current diagnostic work-up of severe febrile illness that might consequently improve treatment choices. However, despite this recognized theoretical potential, implementation of this prospective device in DR Congo is likely to encounter several hurdles, in part related to required financial resources but also to challenges associated with the broader context of febrile illness management.
